# Eliminating Rabies in Tanzania? Local Understandings and Responses to Mass Dog Vaccination in Kilombero and Ulanga Districts

**DOI:** 10.1371/journal.pntd.0002935

**Published:** 2014-06-19

**Authors:** Kevin Bardosh, Maganga Sambo, Lwitiko Sikana, Katie Hampson, Susan C. Welburn

**Affiliations:** 1 Centre of African Studies, School of Social and Political Science, College of Humanities and Social Science, The University of Edinburgh, Edinburgh, United Kingdom; 2 Division of Pathway Medicine and Centre for Infectious Diseases, School of Biomedical Sciences, College of Medicine and Veterinary Medicine, The University of Edinburgh, Edinburgh, United Kingdom; 3 Environmental Health and Ecological Sciences Thematic Group, Ifakara Health Institute, Ifakara, Morogoro, Tanzania; 4 Boyd Orr Centre for Population and Ecosystem Health, Institute of Biodiversity, Animal Health and Comparative Medicine, College of Medical, Veterinary and Life Sciences, University of Glasgow, Glasgow, United Kingdom; The Global Alliance for Rabies Control, United States of America

## Abstract

**Background:**

With increased global attention to neglected diseases, there has been a resurgence of interest in eliminating rabies from developing countries through mass dog vaccination. Tanzania recently embarked on an ambitious programme to repeatedly vaccinate dogs in 28 districts. To understand community perceptions and responses to this programme, we conducted an anthropological study exploring the relationships between dogs, society, geography and project implementation in the districts of Kilombero and Ulanga, Southern Tanzania.

**Methodology/Principal Findings:**

Over three months in 2012, we combined the use of focus groups, semi-structured interviews, a household questionnaire and a population-based survey. Willingness to participate in vaccination was mediated by fear of rabies, high medical treatment costs and the threat of dog culling, as well as broader notions of social responsibility. However, differences between town, rural and (agro-) pastoralist populations in livelihood patterns and dog ownership impacted coverage in ways that were not well incorporated into project planning. Coverage in six selected villages was estimated at 25%, well below official estimates. A variety of problems with campaign mobilisation, timing, the location of central points, equipment and staff, and project organisation created barriers to community compliance. Resource-limitations and institutional norms limited the ability for district staff to adapt implementation strategies.

**Conclusions and Significance:**

In the shadows of resource and institutional limitations in the veterinary sector in Africa, top-down interventions for neglected zoonotic diseases likes rabies need to more explicitly engage with project organisation, capacity and community participation. Greater attention to navigating local realities in planning and implementation is essential to ensuring that rabies, and other neglected diseases, are controlled sustainably.

## Author Summary

Mass vaccination of dogs is the most effective strategy to eliminate dog-mediated human rabies from developing countries. In 2009, a large-scale elimination demonstration project was funded and coordinated by the Bill and Melinda Gates Foundation (BMGF) and the World Health Organisation (WHO) in three southern countries, including the United Republic of Tanzania. This paper explores community perceptions and responses to this programme in the districts of Kilombero and Ulanga, Southern Tanzania. The study was based on focus groups and interviews in 16 villages as well as a household questionnaire (n = 113), a population-based survey (n = 6,157 households) and key informant interviews (n = 24). The study showed that fear of rabies, the threat of dog culling and broader ideas of community responsibility drove compliance. However differences in local livelihoods shaped dog ownership patterns and the distribution of dogs in ways that were not explicitly addressed by project strategies. A survey in six villages found that only 25% of dogs had been vaccinated in 2011. We discuss the operational constraints and problems that lowered coverage as viewed by different actors at the district and village-level. A more explicit engagement with project organisation, capacity and community involvement are needed to address this low coverage.

## Introduction

Rabies has been known since antiquity as one of the most feared human diseases [1]–[3]. Today, it remains a significant albeit neglected disease, causing some 55,000 deaths each year, predominately among children and the rural poor in Asia and Africa [4]–[6]. Transmitted by saliva from the bite of an infected animal, the rabies virus invades the central nervous system and, in the absence of post-exposure prophylaxis (PEP), is fatal once clinical signs appear [7]. Symptoms can be nonspecific but often include hydrophobia, hypersalivation, respiratory difficulties, biting and aggression. Although all mammals can be infected, the vast majority of human rabies cases are caused by domestic dogs [8].

Canine rabies has been eliminated from most industrial economies. In Great Britain, this was achieved in 1902 through a combination of dog licensing, muzzling, culling, tracing movements of rabid dogs and their contacts, and strict quarantine, which continues to be upheld by “pet passports” [3]. However, dog vaccination is now regarded as the most effective control strategy combined with secondary roles for population control, movement regulations and the promotion of responsible dog ownership [8]–[10]. There is a strong economic argument for dog vaccination, as eliminating infection from dogs should reduce the demand for costly PEP [11]–[12]. Yet dog vaccination remains under-prioritised by most developing countries with competing health issues and limited resources. Perceptions held by policymakers are that operational constraints (a lack of knowledge about the dog population, inadequate resources and wildlife transmission) are barriers to vaccination [8]. These perceived barriers may be “overstated and erroneous” [9] as a number of successful initiatives have been implemented. Since the 1980s, for example, a combination of intensive canine vaccination and surveillance efforts in Latin America has shown dramatic progress [13]. However rabies has been increasing in parts of Asia and Africa and remains widespread in over 80 countries [14]. Recently, a number of initiatives have been undertaken [15]–[18], bolstered by new elimination targets set by the World Health Organisation [19].

Rabies is endemic in Tanzania with an estimated 1,500 deaths each year [20]. Two decades of research in northern Tanzania has generated important epidemiological insights while demonstrating that the disease can be controlled [5], [11], [21]–[22]. Tanzania was among three countries selected by the WHO for large-scale rabies elimination demonstration trials between 2009 and 2013 funded by the Bill and Melinda Gates Foundation (BMGF) (see: http://www.who.int/rabies/bmgf_who_project/en/index.html). This represented a shift from a localised research project towards an integrated government programme managed by the WHO country office and implemented by government ministries. This ongoing project stretches over 28 districts in Dar es Salaam, Lindi, Morogoro, Mtwara, Pwani and Pemba regions with a diverse population of over 6 million people and an original estimate of 400,000 dogs. The project comprised annual free dog vaccination campaigns, free supplies of PEP to rural health clinics, and improved surveillance for five years in each district. After the project, dog vaccination was to be institutionalised within the Tanzanian government, who would then pay for maintaining successes and scaling-up activities to other areas of the country as part of a sustainable country-wide programme. The project aimed to demonstrate the feasibility of rabies elimination in a sub-Saharan African context with a strong focus on country ownership, envisioned to help catalyse the development of national programmes in other countries.

To successfully eliminate rabies, vaccination must reach at least 70% of a dog population over consecutive years [14]. Vaccination rates lower than 30% are considered a “waste of resources” [8]. Vaccination coverage declines rapidly in dog populations with high turnover rates [23]. Most dogs in Africa are owned by a family but are free-roaming and generally quite young; some studies show that half of dogs are less than one year of age [24]–[27]. Validated estimates of dog populations are mostly lacking; a recent study in Iringa district, Tanzania showed that the dog population was six times larger than official estimates [25]. However, such estimates are essential for planning successful mass dog vaccinations.

Despite the feasibility of rabies elimination, most vaccination efforts in Africa have failed to achieve high levels of coverage [8]. Interventions are clearly influenced by local dog ownership practices. For example, attitudes towards dogs and the ability and willingness of owners to handle their dogs; the location of vaccination points; and the extent of information dissemination and knowledge of rabies have all been shown to influence compliance [15], [28]–[30]. Dog owners have not been willing to pay the full costs of vaccination, indicating that rabies control should be considered a public good [31]. Central points are not sufficient in some settings; despite higher costs, house-to-house strategies were needed to achieve 70% coverage in more dispersed pastoralist communities in Northern Tanzania [21]. Whilst dog-owner characteristics are important in understanding project outcomes, the capacity and working norms of implementing organizations also play central mediating roles. Although planned at the central level, most campaigns are delivered through (sub-) district-level livestock field officers who mobilise dog owners to attend central vaccination points. Due to the legacy of structural adjustment on the veterinary sector, the state's capacity in animal health is generally limited in much of Africa [32]. Large and remote geographical areas together with low salaries, insufficient resources and rigid bureaucratic norms can further inhibit such campaigns which depend, to a large degree, on adapting strategies to fit community needs [33].

Hence there are risks that new large-scale rabies control programmes in Africa will encounter fairly stereotypical challenges of “top-down” public health interventions in developing countries, known to overlook critical social, cultural, political and economic contexts that mediate effectiveness [34]–[36]. The ethnographic literature is replete with examples of how otherwise efficacious biomedical interventions fall afoul due to divergences in, among other things, issues of power, knowledge, interests and social norms between different social groups; for example, polio in Nigeria [37], schistosomiasis and soil-transmitted helminths in Uganda [38]–[39] and lymphatic filariasis in Tanzania [40], tuberculosis in Nepal [41] and avian influenza in southeast Asia [42]. Furthermore, recent work recognising the interrelationships between social and ecological contexts and drivers in infectious disease control (i.e. One Health and EcoHealth) [43]–[44] as well as the complexities of fostering equitable access to health technologies for the poor (issues of acceptability, adequacy, affordability, availability and organisational architecture) [45]–[46] have highlighted the need for programmes to better understand and engage with the key “effectiveness determinants” that mediate outcomes.

It is increasingly imperative, therefore, that Neglected Tropical Disease (NTD) research explores the perceptions and responses of communities and frontline health and veterinary workers to interventions in order to critically analyse their impact and help tailor programmes for sustainability [47]–[48]. Previous studies examining dog vaccination coverage have been largely quantitative and focused on demographic and spatial factors affecting coverage as well as behavioural characteristics of individual dog owners. To date, there have been no studies detailing how the perceptions, behaviours and contexts of different local actors influence such campaigns, as promoted by actor-oriented perspectives in sociology and anthropology [36]. This article reports the first mixed method anthropological study on canine vaccination in Africa, focused on the predominately rural areas of Kilombero and Ulanga districts in Southern Tanzania.

## Methods

### Study Area

**Figure 1:**
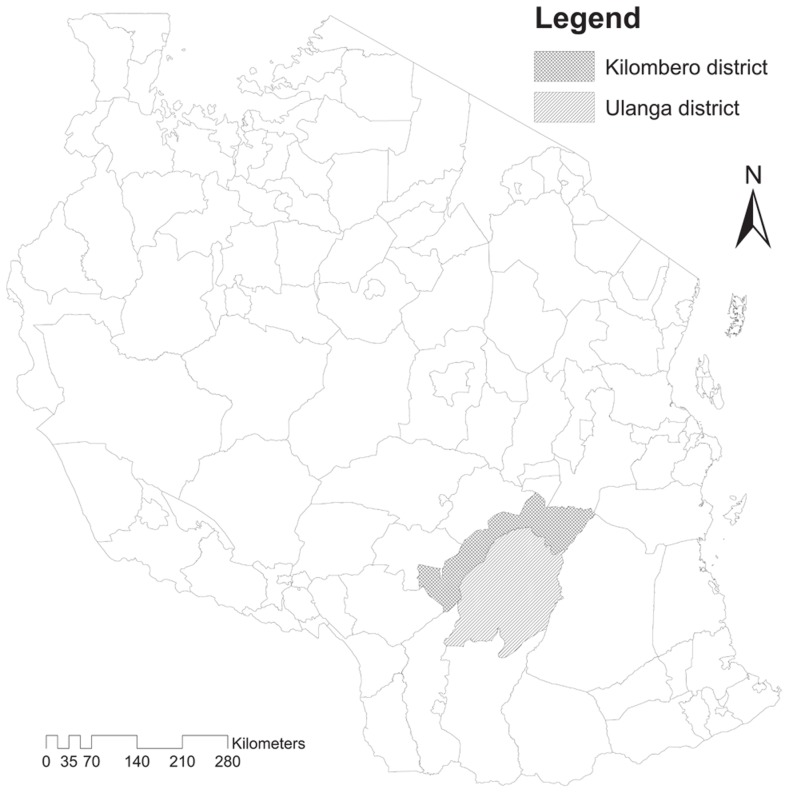
Study districts.

Research was conducted in Kilombero (14,918 km^2^) and Ulanga (24,560 km^2^) districts in Morogoro region, Southern Tanzania, during the dry season from May-August 2012 (Figure 1). These districts are surrounded by the Udzungwa Mountains National Park and the Selous Game Reserve and are roughly divided by one of the largest wetland areas in Africa, the Kilombero Valley ecosystem. The rainy season begins in early November and ends in May. Occasional dry spells from December to March ameliorate flooding that disrupts road transport in the Kilombero Valley during the rainy season. A large diversity of ethnic groups have come to inhabit the area during several historical migrations, include the Ndamba, Pogoro, Mbunga, Bena, Ngoni, Ngindo and Hehe, who speak their local languages as well as Kiswahili [49]. People depend heavily on the natural environment for water, wood, pasture, bush-meat and farming.

The economy of the Kilombero Valley is structured around the farming of rice and maize, livestock keeping, small business, fishing and casual labour. There are also a few large plantations of sugarcane, rice and teak and other formal employment in urban areas, including the district centres Ifakara and Mahenge. Religious affiliation is roughly 40% Muslim and 60% Christian. In 2006 some 657,899 people resided throughout 146 villages within the two districts, with a much higher population density in Kilombero than Ulanga [50]. The area lacks tarmac roads outside the district capitals as well as easy access to a national highway (travel to Ulanga requires the use of a motorised ferry connected to Kilombero), which has certainly helped maintain the areas relative economic and political marginalisation despite its abundant natural resources.

Importantly, dog vaccinations had been conducted in Kilombero and Ulanga for two years prior to the WHO/BMGF project by local researchers following a rabies outbreak in 2007. This was unique among the 28 WHO/BMGF project districts, which had only commenced district-wide vaccinations in 2010; hence our two study districts offered an opportunity to learn lessons about how district teams adapted over time to vaccination campaigns. Implicitly, we assumed that this would translate into improved planning, education, engagement with community needs and understanding of the local dog population as compared to other districts in the project.

### Methods

**Figure 2:**
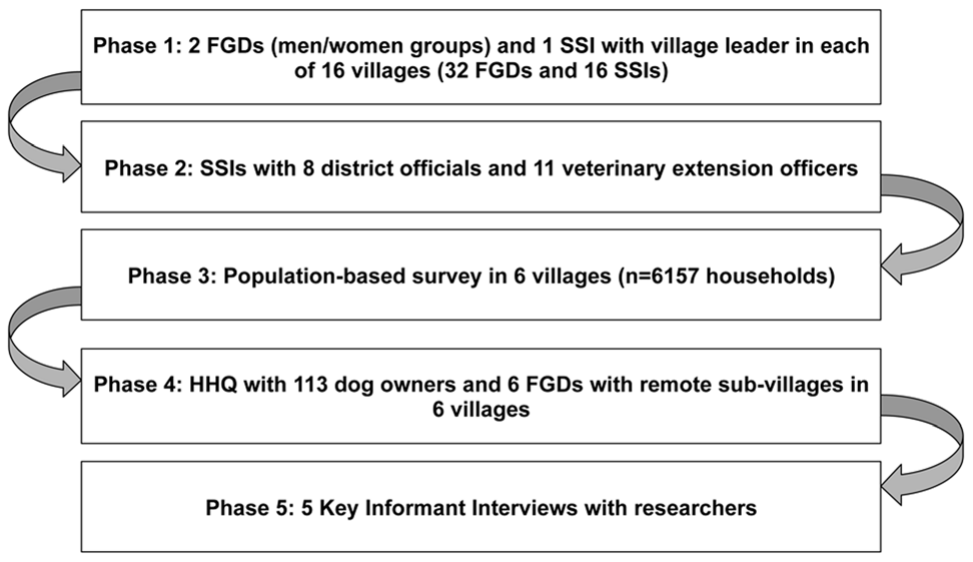
Phases of fieldwork.

The study involved five phases of fieldwork (Figure 2). The first involved focus group discussions (FGDs) with separate groups of women and men (between 6 to 15 people) in 16 villages (8 villages in each of the two districts). These participants were selected in collaboration with the village office to contrast differences in socio-economic status, deliberatively mixing wealthier, middle and poorer participants and those with and without dogs. Semi-structured interviews (SSIs) were also individually conducted with each village leader to clarify details and explore related topics. These interviews and focus groups explored people's knowledge and experience of rabies, attitudes and opinions of the vaccination campaign and dog management practices and attitudes towards dogs. These villages were chosen to incorporate a range of estimated vaccination coverage and known rabies cases (provided by district officials) and included those villages with the most known cases of human rabies and those with no reported cases. Villages were also selected to maximise differences in demographic, cultural and geographical variation, as based on the knowledge of local researchers.

In the second phase, semi-structured key informant interviews were conducted with senior district officials in the medical (3), veterinary (3) and agricultural sectors (2) as well as with 11 livestock field officers responsible for vaccination.

The third phase involved selecting six of the 16 villages originally visited for more in-depth study. Careful attention was given to maximising common variations that emerged from the focus group data, including differences in coverage, rabies cases, livelihood patterns, social characteristics, geography and dog density and management. A population-based survey was conducted in these villages where enumerators visited every household to gather data on the human and dog population as well as vaccination status of dogs and reasons for non-compliance. A total of 6,157 households were found and spot checks of 20 households per village were conducted to verify the accuracy of this data.

Fourth, a detailed household questionnaire (HHQ) with both open and closed ended questions was done with approximately 20 dog owners in each of these six villages (n = 113). Most rural villages were large and dispersed with upwards of 10–20 km in diameter and composed of four to eight sub-villages; hence questionnaire administration was divided equally between the different sub-villages (ranging from four to eight) of each village where an effort was made to seek out households in the most remote and dispersed settlement areas. This questionnaire explored livelihood characteristics, dog management, disease knowledge and attitudes towards vaccination. Since residents from remote sub-villages were often few in the initial focus groups, clarification of their experiences was necessary and one focus group was then done with community members (half were male, and half were mixed gender, groups) in the most remote areas of each of the six villages on similar topics to those described above.

Lastly, five key informant interviews were done with researchers involved in rabies control in Tanzania to better contextualise the study.

For qualitative data collection verbal informed consent was obtained from each research participant while for quantitative data collection written consent was used. All data collection, except for key informant interviews, was conducted in Swahili and translation from and into English was done. All questionnaire data was entered and analysed using Excel (Microsoft Office Excel 2007). Qualitative data was entered into Microsoft Word and analysed manually. Ethical clearance was obtained from Sokoine University of Agriculture in Tanzania (Ref: RPGS/R/8VOL XI).

## Results

### Vaccination Coverage in Kilombero and Ulanga

As an intervention, 70% coverage of the dog population is needed over consecutive years for rabies vaccination to be successful, making a good knowledge of the dog population essential to planning and estimating coverage. Interviews with the District Veterinary Officers (DVOs) of the two districts showed that the dog population was not well documented. Available data from Kilombero included the 2002 census that reported 21,941 dogs and an informal estimate given by the DVO that this had *“now gone up to about 29,000 dogs.”* For Ulanga, this included a 2009 census that showed 7,385 dogs. Based on the 2006 human census estimates, this would give a human-dog ratio of 12.3∶1 in Kilombero and 28.7∶1 in Ulanga. These are both relatively low estimates compared to other published studies [9]. Other studies in Tanzania in both coastal and inland regions estimated a human-dog ratio of 14∶1, albeit inland rural areas (like Ulanga and Kilombero) had a much higher ratio [30]. Work in the Serengeti among pastoralist and agro-pastoralists showed a ratio of 6.3∶1 [11] and 7.3∶1 [21], while a recent study in a Tanzanian city (Iringa) found a 14∶1 ratio, six times larger than the official district records [25].

Dog registers kept in the DVOs office indicated the name of the owner of each vaccinated dog, allowing for tentative estimates of coverage. For the DVOs, this contributed to estimates of coverage that were far higher than was likely the case: the DVO of Kilombero cited 75% then reduced it to *“at least more than 50% for sure”* with some reluctance, while the DVO of Ulanga stated that *“at least 90% of the dogs in the district were vaccinated, certainly not less!”* However, that rabies was still present (discussed below), albeit reduced from the 2007 outbreak levels, should have been indicative of a much lower coverage, at least for Ulanga. This is especially the case given that rabies oscillates between endemic and outbreak scenarios [51]. Using the official dog population estimates provided by the DVOs and the 2011 vaccination data from their offices, vaccination coverage for 2011 was 40.5% in Kilombero and 102% for Ulanga, with lower figures for 2009 and 2010 (see Table 1). Unlike with Kilombero where routine vaccination was also done, dogs were only vaccinated in Ulanga during the campaign as the district lacks the necessary cold chain outside the district capital.

**Table 1:** Official vaccination coverage.

District	Dogs vaccinated 2008	Dogs vaccinated 2009	Dogs vaccinated 2010	Dogs vaccinated 2011
Ulanga	31% (2,278)	100% (7,385)	50% (3,676)	102% (7,555)
Kilombero	None	18% (5,178)	31% (9,073^1^)	40.5% (11,746^2^)

Source: District veterinary office, Kilombero and Ulanga districts.

^1^ In 2010, the campaign lasted 11 days in Kilombero and vaccinated 7,639 dogs while 1,434 dogs were then vaccinated during routine vaccination.

^2^ In 2011, this included 9,194 dogs vaccinated in Kilombero during a five day campaign and 2,552 dogs vaccinated during routine vaccination.

In discussions with government officials and villagers it became clear that there were very different assessments of how successful the vaccination campaigns had been. Apart from the low coverage in Ulanga in 2010 (explained below), government perceptions emphasised that coverage had been increasing in parallel with the experience of the extension officers, the addition of more central points, the involvement of teachers, nurses and doctors during the campaign, and greater practice and trust with dog-owners. The high coverage reported by DVOs was reiterated by the 11 interviewed livestock field officers (LFOs), most of who had been involved in all three or four campaigns. Despite some scepticism that 70% of dogs had been vaccinated, not one believed less than 50% had been vaccinated with most placing the estimate at 60% and some more than 80%. In contrast, focus groups and interviews with community members emphasised the small proportion of vaccinated dogs, placing their own unofficial estimates between 25 to 50% coverage.

### Livelihoods and Dogs

Understanding vaccination coverage requires considering the various links between livelihoods and dogs in Kilombero and Ulanga, which varied greatly between social groups with important implications. While there were other common uses for dogs (hunting, companionship, symbols of wealth, to ward off spiritual forces and act as capital assets when selling puppies) by far the most important involved security −97% of questionnaire respondents stated so. However, the particularities of how dogs were used for security, the human-dog relationship and how dogs were managed differed between cattle keepers (both agro-pastoralists and pastoralists), rural farmers and town residents.

For farmers (the majority of the rural population), dogs represented a “line of defence” between crops and certain destructive wildlife. For example, 86% (n = 97/113) of questionnaire respondents claimed to suffer from varying degrees of wildlife encroachment on their farms. While elephants and buffalo could cause major damage, these were rare and monkeys and baboons were the major problems where they, in the words of one angry woman, *“finish off a large portion of my crop in one day and enjoy harassing our maize the most.”* This was further impacted by the geography of the farm relative to the homestead, forests and wetlands. Following the villagization programme of the post-independence era in Tanzania [52] as well as the need to cultivate rice in the wetlands, homesteads were often far away from farms. During the growing season, farmers either migrated from the village to a small makeshift hut for a few months or commuted daily from their homes, with many taking their dogs with them. Wildcats, mongooses and jackals were known as thieves of chickens and chicken eggs and dogs were also commonly kept to protect them.

Reasons for keeping dogs were different for livestock keepers. For Masai and Mang'ati pastoralists and the agro-pastoralist Sukuma ethnic group, dogs were used to guard cows, goats and other livestock during grazing and in the cowshed at night from thieves as well as wild dogs, jackals, hyenas and the occasional lion and leopard. In small groups sometimes with hundreds of cattle, young men migrated between the village outskirts and the wetlands and forests following pasture and waterholes during the dry season. These were generally either a short one or two hour walk away from the home (if routes did not infringe on farmland) or large distances of upwards of 20 km or more. Women, children and elders would remain resident in the village during these migrations, most often in remote and dispersed sub-villages far from main access routes. Pastoralists were considered (and observed) to own many more dogs than farmers and their dogs were also bigger, more aggressive and more loyal and alert. Long migration routes as well as cultural determinants (i.e. emphasising a “warrior” attitude, common to these pastoralist groups) cultivated closer bonds between dogs and male (agro)-pastoralists than with most sedentary farmers.

This contrasted with the small or large town centres dispersed throughout the area where thieves were the main rationale for dog ownership. There were a number of reasons given for why dog populations were considered far smaller in more densely populated areas: land owners not permitting the keeping of dogs; the higher chance that town dogs would cause conflict by biting people in the street; an idea that urban residents were more “educated” and would keep fewer dogs; and urban residents reporting that they practiced “proper” Islam that restricted the keeping or touching of dogs. According to certain Koranic rules, these groups emphasised that physical contact with a dog (saliva and fur) would make someone spiritually unclean (especially before prayers). For these reasons, Muslims in towns stressed that, although they could keep dogs, they had to *“treat them well as Mohammed said…and have them only for a specific purpose.”* Regardless of religion, urban dogs were believed to be better cared for and more likely to be vaccinated than dogs in rural areas, with a few confined to their household (unlike the vast majority of dogs that were free roaming).

Therefore, differences in livelihood patterns (and their culturally-embedded dynamics) between town, farmland and pastoralist systems influenced the human-dog relationship and the spatial distribution of dogs in Kilombero and Ulanga. Utilitarian value tended to mediate and dictate dog management rather than purely culturally-defined beliefs and practices. This clearly impacted vaccination coverage rates: villages that believed vaccination coverage was highest were from more urban areas situated along main roads but with fewer dogs, whilst lower coverage estimates were given in those villages in more remote areas, known to have higher dog populations.

### Local Knowledge of *Kichaa cha Mbwa*

Local knowledge of rabies also revealed a general perception of low vaccination coverage, reflected in understandings of rabies epidemiology, experiences of rabies cases and attempts by village leaders to institutionalise “village laws” in order to address non-compliance. Rabies was linked to its Kiswahili name *Kichaa cha Mbwa* (madness of dogs) and widely known as a fatal disease of dogs and humans that affected the brain, was transmitted by animal bites and prevented by dog vaccination, similar to a recent large-scale questionnaire study in Tanzania [53].

Aside from this basic knowledge, rabies was considered an “outbreak disease”, understood in relation to four interrelated beliefs. First, it was a disease of “dirty dogs” caused by neglected (but owned) free roaming dogs that spread the disease due to poor animal welfare and poverty. This narrative emphasised that although most farmers and town residents claimed to own dogs for security, this was often an assumed rather than actual use. Many dogs were considered lazy, not aggressive enough, unable to be trained and always away from home looking for food or a dog of the opposite sex. They lacked a clear utilitarian value, which in turn fostered “negligent owners” who did not care for their animals and, therefore, facilitated the spread of rabies. In the words of one village leader, “*living as we are in this farming environment [as poor farmers], dog owners keep dogs without a purpose and do not care about them so they move all over the place…and this is how they catch rabies.”*

The second common narrative involved the idea that rabies had never been a problem in the Kilombero Valley until the migration of Masai and Sukuma from northern Tanzania imported rabies as they moved into the area in the late 1990s, which strengthened animosity between farmers and (agro-) pastoralists in certain areas [54]. Third, rabies was believed to spread from wildlife to dogs, facilitated by farmers, hunters and pastoralists living near game reserves and national parks and influenced by seasonal changes in rainfall affecting the movement of carnivores. Lastly, rabies incidence was considered to increase during the harvest period in June and July corresponding with the mating season.

The majority of people approved and understood the role of canine vaccination. Differences between biomedical and local understandings, known to lead to community resistance to other human and animal vaccination programmes [37], [55], were largely absent. Although rumours that the vaccines were killing dogs and that the campaign was a government dog culling programme had been widely disseminated during the 2008 and 2009 campaigns (before the WHO project), these concerns had abated with time and side effects to vaccines (real or perceived) were rarely mentioned.

Part of this had to do with the high level of awareness about rabies, underpinned by local experiences of human cases. Although open to error, focus group participants and village leaders identified (with detailed symptoms and related circumstances) a total of 59 suspected rabies death cases in the 16 study villages within memory, most (45) reportedly from 1995 to 2008, but with four deaths identified in 2012 (the year of field research). While most were from dogs, there were a few attacks from jackals and wild dogs. This would give an average of 3.2 cases per year (1995–2008) in these 16 villages (population 30,143), implying 10.7 cases/100,000 people; much higher than the 4.9/100,000 estimated for the country as a whole based on active surveillance in Northern Tanzania (this difference can be attributed to the fact that our selected villages included those most affected by the outbreak between 2007–2008) [20]. Contact tracing as part of a related research project (where researchers follow up all reported suspected rabies bite cases) showed 30 deaths in the two districts since 2007, with most prior to 2009; in turn, hospital records between 2009 to mid-2012 showed 478 bite victims of suspected rabid animals divided equally between the two districts, with only 2 reported deaths (Unpublished data).

These local accounts of having neighbours and relatives die from rabies or have to seek treatment after being bitten by a dog generated a significant degree of fear and apprehension. This clearly motivated many households to comply with vaccination. Asked if they would prefer acquiring HIV/AIDS or rabies, 33% of questionnaire respondents picked HIV/AIDS while 14% could not choose between the two. While people mentioned hydrophobia, muscle spasms and nervous twitches, they stressed that respiratory symptoms made victims *“bark”* like the animal that had transmitted the disease: rabies made people *“act like wild animals”* and *“die like mad dogs.”* They became *“demon-possessed”*, started to *“bite everything”* and become *“so strong like the animal that bit them.”* Furthermore, access barriers to treatment (high costs and inadequate access to medicines and health services more generally) drove community fears. As one woman stated, *“For rabies, if you are bitten today and cannot get treatment, which is so common here, tomorrow you die like an animal”* (Focus group participant, Sanje village, Kilombero).

This level of fear drove communities to attempt to institutionalise two different “village laws” in order to increase compliance with vaccination and deal with suspected rabid dogs and bite victims. In response to the 2007 outbreak and recent vaccination campaigns, most villages had established local bylaws indicating that dog bite victims should be financially compensated for medical costs by the dog owner if the dog was not vaccinated; albeit compensation was never guaranteed. Some never pressed their neighbours for payment, others were not able to identify the dog owner, and others were not able to prove (in the village court) that the accused dog actually belonged to the owner (given the lack of records) or was not vaccinated (certificates could be used interchangeably between dogs). Second, there were various endogenous attempts to standardise dog culling after vaccination, considered an ethical and effective method of rabies control at the village-level (but in no way promoted by the WHO project). In many villages killing unvaccinated dogs was considered a “district law” with support from livestock field officers; albeit the passing of the Animal Welfare Act (2008) made this law ambiguous. The most common suggestion to improve coverage was for the village office to require dog owners to register their dogs so that after a vaccination campaign, a grassroots “local committee” could move house-to-house eliminating unvaccinated dogs (evident by the lack of a new collar and the vaccination certificate). This was often done by villagers themselves in haphazard ways that led to protests from dog-owners. Responses to dog bites (despite many caused by aggressive dogs, bitches with puppies, dogs defending their homestead from strangers or provocation) were always treated as suspected rabid cases and involved quickly killing the dog, and often provoked a spontaneous dog culling spree.

The importance of strengthening these two endogenous attempts to enforce dog vaccination was ubiquitously emphasised, reflecting local perceptions that the rabies control project was achieving low-levels of coverage. During focus groups and interviews, the relationship of rabies to “negligent” dog owners, pastoralists, wildlife and seasonal variation quickly veered into discussions about how vaccination campaigns was not sufficiently addressing what were considered key points for controlling the virus; there was a need to better prioritise targeting households bordering wildlife populations, synchronise vaccination with the farming season and pastoralist migrations, and motivate the many “negligent dog owners” through recourse to village laws and punishments, supported by district authorities more systematically. But how many dogs were truly being vaccinated?

### Estimating Coverage: Population-Based Survey

Given the divergent views of government officials and villagers, there was a need to generate more robust estimates of the dog population and vaccination coverage; hence, we carried out a population-based survey in six selected villages. The survey showed that out of a total of 6,157 households and 30,143 people, there were 1,311 dog-owning households (21% of households) and 3,056 dogs (Table 2). This included 2,414 dogs older than one year and 642 dogs less than one year. While this gave a total human-to-dog ratio of 9.86∶1, this was highly skewed following local knowledge that the dog population was predominately in rural and remote areas. The more urban villages (or towns) of Mwaya and Chikwera had a human-to-dog ratio of 31.4∶1 and 64∶1 while the rural villages of Mofu and Namhanga had ratios of 6.9:1 and 5.8∶1. However the low population in Mwaya was also a consequence of mass dog culling campaigns that had taken place in 2008 and 2010 in response to human rabies cases. This variation was equally pronounced within each of these villages. For example, sub-villages bordering forests in Machipi and Mwaya had a much higher human-to-dog ratio than other areas. Likewise, the sub-villages with pastoralists in Namhanga and Signali had double, and in Mofu village more than 10 times, more dogs compared to other sub-villages but with relatively equivalent human populations. This showed that the dog population was highly skewed even within individual villages, based on surrounding ecological characteristics that influenced dog utility.

**Table 2:** Vaccination coverage in six villages.^1^

District	Village	People	Households	Households with dogs	Dogs	Vaccinated dogs	Dogs not vaccinated	Dogs less than one year	Vaccination coverage^2^
Kilombero	Signali	4508	606	132	390	116	274	122	30–43%
	Machipi	1955	461	116	233	117	116	37	50–60%
	Mofu	8375	1550	640	1433	265	1164	186	19–21%
Ulanga	Mwaya	6055	1491	98	193	34	159	66	18–27%
	Namhanga	5125	912	297	742	208	534	213	28–39%
	Chikwera	4125	1137	28	65	29	34	18	45–62%
	**TOTAL**	**30,143**	**6,157**	**1,311**	**3.056**	**769**	**2,287**	**642**	**25–32%**

^1^ Village specific coverage data was only available from Kilombero. This showed 123 dogs vaccinated in Signali, 284 in Mofu and 72 in Machipi. The small discrepancy between these figures and the survey data can be attributed to death of vaccinated dogs, poor record keeping, changes in dog ownership, vaccination of dogs from other villages as well as response bias in the survey.

^2^ Coverage range that includes all dogs at the time of the survey (lower estimate) and excludes all dogs acquired after the 2011 campaign (higher estimate).

Furthermore, the population-based survey also confirmed the low coverage emphasised by community members. In total, only 769 dogs (25% of the canine population) had been vaccinated in 2011, whereas 2,287 dogs (belonging to 923/1,311 households) had not been vaccinated. If the 642 dogs born since the vaccination campaign (21% of the dog population) are excluded, coverage rises to 32% of the mature dog population. The immunised population is slightly lower given the small percentage of stray dogs; however, this is a relatively negligible population given scarce food resources, estimated at 3–5% in rural northern Tanzania and 1% in urban areas of Iringa, Tanzania [11], [21], [25].

As with dog density, vaccination coverage also varied between villages (Table 2) with the highest coverage in both Machipi and Chikwera villages and lower coverage in Mofu and Mwaya. Importantly, dogs in the low coverage villages of Mofu and Namhanga together accounted for 71% of the total dog population of the six villages (with 2,175/3,056 dogs) due to settlements of pastoralists and remote farmers in a number of sub-villages, which were far from main access roads. In contrast, the two villages with highest coverage rates included a large town with only 65 dogs (Chikwera) and a village (Machipi) relatively close to the district capital in Kilombero.

### Barriers to Canine Vaccination

**Figure 3:**
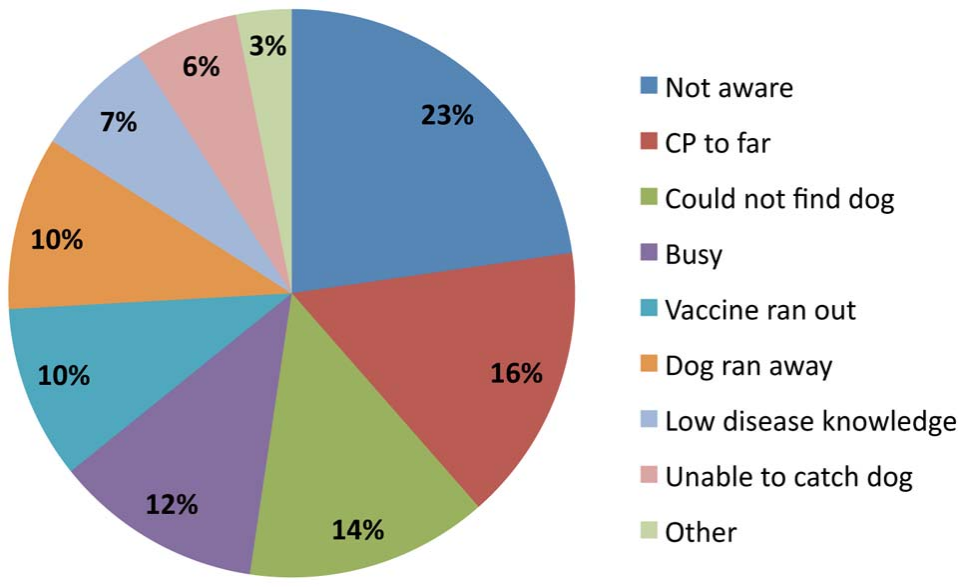
Reasons given for non-compliance with vaccination. Results from the population-based survey, including n = 750 household respondents without vaccinated dogs considered old enough for vaccination.

While most people understood the role of canine vaccination, interrelated geographic, social and operational factors created a number of important access barriers. In the population-based survey, reasons given by the 750 dog-owning households (with dogs born before the 2011 campaign and considered eligible for vaccination) for non-compliance included (in descending order of importance): not being aware that the campaign was taking place (23%), having a central point too far from their homestead (16%), not being able to find their dog (14%), not being available that day (12%), the vaccine having run out (10%), having the dog run away during transport or at the central point (10%), not being aware of the importance of vaccination (7%), not being able to catch the dog (6%), having a young puppy or pregnant female (2%), a perception that the vaccine has side effects (1%) and having just recently moved to the area (0.2%) (Figure 3). However, understanding how and why these various barriers existed requires triangulating this with qualitative data.

#### Mobilisation and information dissemination

Contact between district officials and villages began with mobilisation: disseminating information about the time, place and purpose of the campaign. Letters were sent to village offices, radio announcements made and posters put up one or two weeks prior. In some areas, meetings were held between LFOs and villagers and announcements made in schools, churches, mosques and public areas. The day before the campaign, announcements were also commonly made with drums or a loudspeaker mounted on a car.

Opinions differed dramatically over the extent, timing and impact of these efforts. The village officers, who were sometimes given *“some little money”* for motivation by LFOs, were tasked with much of the mobilisation. However, villages were often large and composed of a number of sub-villages far from the village office and most formal announcements were focused solely around access routes, shops and the village office. LFOs relied on the village office to use sub-village leaders (as well as schools, mosques and churches) to reach other areas but without any financial incentives and an often short notice provided either by the LFO or the village office (many times given the night before) mobilisation was done poorly. This explains, to some degree, why “not being aware that the campaign was taking place” was responsible for 23% of dog owners not participating in the 2011 campaign.

#### The timing of the intervention

It was a common complaint by community members that, in the words of one local leader, *“We find that the LFOs structure the day of the intervention around district officials and not the recipients.”* While it was natural that some households were busy, there were a few villages where local markets were not accounted for by the LFOs. Furthermore, vaccination often ended either before or just after school finished, creating challenges for children in vaccinating the household dog.

The most significant aspect of timing, however, involved the month of vaccination in relation to bureaucratic norms and the migration patterns of pastoralists and some farmers. Pastoralist herders (together with most of their dogs) were often away from the village during the dry season from July/August to November/December, depending on the rains. Some farmers, in turn, had been busy preparing their farms for the approaching rains at the beginning of October (just before the 2011 campaign) having already migrated to their farmland. Hence there was a clear divergence between the needs of pastoralists (who tended to recommend June as the ideal month for vaccination) and farmers (who recommended August or September). Despite this, district officials had a difficult balancing act since much of Kilombero Valley is flooded from December to May or even June, making numerous areas inaccessible. This was further compounded by the budgetary requirements of the district government whose financial year ends in July. District funds received in one financial year could not be used in the next. In the words of one official from Ulanga:

“*This does not allow us to receive money in June and then plan for the vaccination in September…sometimes we don't even know when the money will be coming so that in 2010 it arrived in June and we had to do the vaccination as fast as we could, even though some areas were still flooded.*”

The fact that the 2011 campaign had been done in mid-October (other campaigns had been done between September and early October) meant that many pastoralists (and some farmers) were away from their village with their dogs.

#### The placement of the central point

Having a central point (CP) too far from the homestead was found to be the principal reason why 16% of households reportedly did not vaccinate their dogs. Community members accused the LFOs of *“not consulting the people”* and wanting *“somewhere comfortable to have [CPs] since they don't want to use fuel to come deep to us in the remote areas.”* Despite the insistence of district officials that vaccination points had been *“chosen by villagers”* most were located at the village office, typically in the centre of the village near roads and shops; others included football pitches, schools and large fields. While this was sometimes sensible, local leaders had clearly chosen the area used for most village activities, despite not always being the most appropriate and well beyond the 500 m or 10 minute walk recommended by the WHO [14].

Some were chosen by considering the number of dogs: of the 16 villages visited for focus groups, eight reported one CP in 2011 while eight had two. Those villages with only one site were more densely populated, such as towns or smaller villages. In villages with two CPs, one was typically the village office while the remaining CP was situated in a remote area. Over successive campaigns since 2008, LFOs emphasised they had improved their ability to target remote sub-villages. One of the reasons why Machipi village in Kilombero had 50% coverage (the highest in the population-based survey) was that the LFO, who lived nearby, located a CP in the most remote sub-village despite requiring crossing a river on a dugout canoe! This clearly shows the importance of having LFOs consult with the village office and sub-village leaders and be willing to adapt strategies to meet local needs. However, there were two difficulties found with this strategy: (i) in a few villages with two central points, the day was merely divided between the two locations limiting the time dog owners in one site could bring their animals; (ii) in others, LFOs demanded a small fee for each vaccination to *“cover fuel charges”*, which significantly reduced compliance.

#### Bringing dogs to the vaccination point

A total of 10% of surveyed households reported that their dog ran away either on the way to, or at, the central point; a further 6% reported that they could not catch the dog. Most dogs were brought to the CP by the father or son. If the dog was considered *“the property”* of the father, a son had to ask permission before vaccinating it – problematic if he could not be reached. Some fathers (with unvaccinated dogs) believed that taking a dog to a vaccination point was embarrassing and were ashamed to since it was considered *“a child's duty.”* Most dogs were free roaming making catching a dog and tying it difficult, and most relied on having their dog(s) follow them without a leash; however many dogs did not listen to their masters. Remote households (especially pastoralists) reported to only take some dogs to the CP since they were not able to handle all of them over long distances crossing densely populated areas (and entering, from a dog's perspective, into *“foreign territory”*). Additionally, the result of having so many free roaming dogs at the CPs invariably led to some dogs fighting and others running away before they could be vaccinated.

#### The “mindset of the people”

The survey showed 7% of households with unvaccinated dogs were “not aware of the importance of vaccination” while 14% “could not find their dog.” This was related not so much to lacking basic knowledge about vaccination but rather to not having *sufficient* motivation to act on it. This was discussed in two interrelated ways. The first emphasised that variations in the human-dog relationship within individual villages (as shown in a recent study in Ethiopia [56]) were heavily influenced by livelihood utility rather than “culture.” Owners who did not have a concrete purpose for a dog were believed to “neglect them” (i.e. lack an incentive to care for the animal and have less of a bond with it) and be less willing and able to vaccinate them regardless of ethnicity. Many of these dogs were acquired by children without parental consent and it was common for such dogs to obtain food from multiple households (and be known as local *“thieves”*) where they were sometimes not seen by their “owners” for a number of days at a time. In contrast, hunters, farmers in need of protection from wildlife and most (agro)-pastoralists had more affectionate feelings towards their dogs and considered participating in vaccination one manifestation of this positive relationship.

The second narrative emphasised the link between dog-owners' motivation to participate in rabies control, risk perceptions and wider socio-developmental issues (i.e. poverty, education and social solidarity). According to district staff, poverty and low education were the main reasons for non-compliance; for example, *“if the government was announcing free maize…everyone would come running* [but since rabies was an] *outbreak disease it requires educated people* [a wealthier and more educated population] *to make everyone act together.”* Some dog-owners candidly admitted that they did not believe rabies was a major problem in their communities and that, since the likelihood of their being bitten by a rabid dog was small, they could not be bothered to take the time to vaccinate their dog(s). To others, this lack of motivation was interpreted as a *“lack of community spirit”* and representative of “*being a disorganised person,”* considered antithetical to community cooperation and development. People generally believed there were more *“negligent”* dog owners than *“organised”* ones, and that this greatly reduced coverage. This was reflected in the prevalent view that more effort needed to be made in strengthening “village laws” on vaccination, the killing of unvaccinated dogs and the mandatory payment of treatment costs to bite victims paid by dog owners (discussed above).

#### Problems of equipment and staff

Although community involvement was believed to be lacking, there were important operational difficulties surrounding equipment and staff that were perceived and experienced in different ways. Sometimes tasked with both livestock and crop extension services, community perceptions of LFOs echoed frustrations with government services more generally, as they were believed to *“not give any of their time in educating the people about proper dog management”*, were *“lazy and not helpful”* and *“preferred staying in their offices than helping the people in the village.”* Villagers felt *“voiceless for our right to have a field officer helping us”* for animal diseases more generally since many areas had no LFO or were part of a very large catchment area. Many had negative perceptions of their local LFOs who, according to one village leader, *“do nothing to register dogs and ensure all dogs are vaccinated, they don't come deep and don't communicate well with the village about when things will take place…those people are useless!”* As one key informant described this in historical context:

“*The project had capacity problems due to the structural adjustment policies since before in the 1970s you had an LFO in every village in Tanzania and they were involved directly with the people but then everything was removed and fell apart. Only now are we trying to improve things but in some areas the communities still do not have much contact with them…they are moving forward but for the rabies project they had no staff, not enough people on the ground.” (Key informant, Tanzania.*)

This echoed complaints by livestock officers about shortages of fuel, staff, vaccines and other equipment. Shortages of fuel were seen as limiting the placement of CPs in more remote places and the provision of sufficient mobilisation. In many instances, community members complained that the lack of staff required them to wait in long lines. Without dog catching equipment, LFOs found it hard to restrain some dogs. As one stated:

“*Sometimes you find that you are only one at the site and rely on a local teacher to be the recorder. The village people don't follow instructions to tie a dog with a rope…handling the pastoralist dogs is very tough…you don't have enough fuel to reach deep into the village and sometimes you even run out of vaccines since you can only carry so much at one time.” (Interview, livestock field office, Kilombero).*

Running out of vaccines was relatively common −10% of households claimed this was why they did not vaccinate their dog(s). Tenuously scheduled follow-up times were sometimes not followed through on by the LFOs themselves which clearly reduced trust with community members. For the LFOs, however, issues of vaccines, fuel and staff shortages were related to a shortage of field funds more generally. This also impacted their own allowances, apparently paid at half of what was initially promised, which clearly de-motivated them.

#### Budget flexibility and project organization

For the district officials in charge of the vaccination campaign resources like fuel and more staff was something that they believed was beyond their control. Rather, this was related to operational budgets determined by the central government and the WHO country office. As one official stated:

“*The WHO rarely involves us in planning or arranging things. In 2009, we prepared budgets but they were rejected since they were too expensive…these people just sit in Dar and pilot things from their desks! But this district is so much larger than other districts in the project, but they do not budget for these differences. They just give the same allocated budget to each district…everything is so fixed…I have no power on the budget, it just comes to me and I am helpless.” (Interview, key informant, Kilombero).*

These officials understood that most dogs were in isolated places (near forests and in pastoralist areas) but felt that they could not budget the appropriate resources to reach them. This lack of flexibility was contrasted with the first round of vaccination in the two districts supported by a research team. Here, according to the same informant quoted above, *“there was adjusting of the budget to address certain problems on the ground.”* Similarly, the problems with the 2010 vaccination in Ulanga relating to the annual fiscal year ending in July (discussed above), reflected issues with budgetary rules and regulations in Tanzania more generally:

“*Once the funds are in the country, you can't access them and there is a lot of bureaucracy…strict rules about how to use funds in relation to the DVO and LFOs. In an NGO setting things are more simple…you give the field staff the money they budget for and if things come up, you adjust it to deal with the problem, but reporting in the government system, you can't do this…if you budget for 2,000 dogs you get vaccines and supplies for 2,000 dogs, nothing more…there are tight budgets and organised procedures.” (Key informant, International).*

In northern Tanzania, rabies research and dog vaccination was “managed like an NGO” based on the ability to be flexible and respond to changing conditions on the ground. It was through this work that the evidence-base for rabies elimination in the Tanzanian context was generated. It was not that the WHO project lacked a budget but rather that the budget had been largely used to supplement the lack of infrastructure in the country. Key informants estimated that upwards of 80% of the budget had been directed towards the per-diems of officials and allowances of field staff since, in the shadows of structural adjustment, field activities required supplementing existing base salaries with additional funds. This was the main reason why the first vaccination campaign in 2008 (done by a research team) had excluded Kilombero district: the district had refused to pay the per-diems of livestock field officers despite freely available vaccines while Ulanga district had managed to find funds.

## Discussion

The feasibility and cost-effectiveness of rabies control and/or elimination through canine vaccination has been well documented, with some noted successes from developing country contexts [9], [11], [13], [18]. However there are clearly challenges in mobilising resources for canine vaccination as well as operational barriers that inhibit success in many contexts. With renewed global attention to rabies following advocacy efforts by the NTD community, there is a need to think critically about how local realities intersect with technical solutions; how should we think about the challenges of dog vaccination for rabies and, importantly, how can large-scale canine vaccination projects navigate local social and ecological complexities in resource-limited settings?

Much recent work in the field of sustainable development and global health (including that of many anthropologists) has emphasised the importance of understanding interventions from the perspective of community-equity effectiveness and using transdisciplinary approaches rather than narrowly emphasising the efficacy of scientific tools and strategies [33]–[48], [57]–[58]. Effectiveness has been conceptualised as a “step ladder” where different variables (at multiple levels) have lesser or more impact on outcomes depending on social, cultural, biological, economic, political and ecological contextual factors [59]. Analytically investigating these “effectiveness determinants” is deemed essential to understand their multiplicative effects. Intervention planners, therefore, are encouraged to identify and engage with high-level determinants, enabling factors and local capacities (that act as essential nodes) in order to move away from managing risk to building resilience and understanding interventions as “complex systems” [44], [60].

Exploring the implementation and community response to a WHO-coordinated canine rabies elimination project in two southern districts of Tanzania, this article has presented (to our knowledge) the first anthropological study of a contemporary dog vaccination programme in a resource-poor country. In the absence of credible estimates, a population-based survey in six selected villages showed that 25% of the dog population had been vaccinated in 2011. The survey quantified what was general knowledge among the village population – that the campaign had achieved coverage well below the 70% target due to a number of interrelated social processes, geographical characteristics and challenges in project implementation. Furthermore, while it is difficult to extrapolate the findings of this study to the wider WHO project area, many key informants believed that Kilombero and Ulanga, due to its prior experience with mass dog vaccinations, achieved relatively high levels of vaccination coverage, suggesting that the difficulties encountered here were not unique. But what were the most important bottlenecks to the canine vaccination project in these two rural districts that had the greatest leverage on mediating intervention effectiveness, and therefore should be most emphasised and reflected on for future vaccination campaigns in Tanzania and elsewhere?

At the community level, there were clear spatial differences in dog distribution driven by the variable dog keeping practices of rural farmers, town residents and (agro-) pastoralists. While dogs played important roles that were embedded within local livelihoods, there were differences between conceived uses and actual ones. Many dogs used “for security” were actually poorly fed and maltreated with little or no clear role in the household. Awareness of rabies, at least on a basic level, motivated people to participate in rabies control out of fear of *“dying like a mad dog”* as well as, to varying degrees, having their dog culled and being held responsible to pay for someone's medical treatment. Equally important were broader notions of social responsibility that reflected much broader divisions within these communities about the willingness to control diseases that were perceived to be relatively rare. Some people in these predominately rural geographies themselves under-prioritised (or neglected) the importance of rabies control given the multitude of other challenges in their daily lives. The widespread emphasis on the need for local bylaws to punish dog owners who did not vaccinate their dogs and monitoring of vaccination status by the village office was a general expression of a desire to motivate (and coerce) non-compliant “negligent” dog owners. Given the difficulties of behavioural change in resource-limited settings [61], there is surely an important role to sustain education campaigns to help increase and facilitate prioritisation at the village-level over the long-term, with a possible role for dog registration.

However, barriers to vaccination did not rest solely, or predominately for that matter, with communities. The rabies elimination project suffered from stereotypical challenges of “top-down” public health programmes. There were critical gaps in communication between central government authorities, district officials, field staff and the target population that were structured by existing bureaucratic procedures, social norms and an over-emphasis on technical solutions. In both districts, an underestimation of the dog population increased what was found to be an erroneous perception of success. The dog population was not geographically uniform but heavily skewed, found largely in more remote areas bordering forests and the outskirts of pastoralist villages, than the more accessible towns or areas with easy access routes. These relationships found expression in local understandings of rabies epidemiology – related to pastoralist migration and wildlife interaction – which were not well incorporated into project planning.

These operational challenges were exacerbated by the long-term effects of structural adjustment policies in the veterinary sector in Tanzania that have significantly reduced the capacity of the state to deal with animal health [32]. This found expression in the negative attitudes of most villagers towards their local livestock field officers; the lack of sufficient fuel, vaccines, staff and “promised” salaries; and the perceived inability of district officials to adjust budgets to address local challenges, such as the large geographical area and the need to adapt the timing of vaccination campaigns to fit seasonal specificities (rainfall and migration). A mixture of lack of funds, planning and capacity as well as the government's financial distribution system prevented flexible, context-specific strategies. As a result, the effectiveness of mobilisation, the location of vaccination points and the timing of the intervention were not optimal. Efforts to increase involvement of community members in mobilisation or to adapt vaccination points based on local recommendations were generally limited by capacity and funds. It was not that local district officials were necessarily oblivious to these challenges; rather they felt unable to communicate effectively with those in Dar es Salaam (Tanzania's capital) with sufficient power to enable flexibility. Communication channels were top-down and learning from past shortcomings, or putting this learning into practice, was generally limited. Some of these challenges contrast with rabies research programmes (i.e. work in the Serengeti) where more capacity and flexibility were believed to have allowed for better targeted campaigns and more community involvement.

Between these different geographical, community and organisational dimensions to the vaccination project, this study shows that, despite many endogenous challenges at the level of the dog-owner, issues of capacity, finances and managerial shortcomings severely lowered coverage by preventing field strategies to be adapted to local realities. The major bottlenecks were not with “community compliance” per say but with how intervention strategies navigated the various structural and behavioural factors that mediated access. This shows the need for a more trans-disciplinary and participatory approach in planning, implementing, managing and monitoring and evaluating rabies control programmes. The findings presented here do not suggest that rabies elimination in Tanzania is unachievable; rather, it points to the need to investigate, consider and take seriously local variations and challenges within the project planning cycle. Robust quantitative data on dog populations and vaccination coverage as well as qualitative implementation research are essential for ensuring that project coordinators have a sound understanding of challenges on the ground.

These issues, however, are not unique to rabies but rather part of a much larger debate about the nature of vertical health programmes in developing countries, top-down strategies and the relationship between expert knowledge, donor-led development projects and poor populations [33]–[43]. Policy narratives and donor-funded projects are often shaped by presenting “quick-fix” technical strategies that can be easily “scaled-up” from local successes within short time periods [62]. Donors demand results that showcase quick-wins, large impacts and “value-for-money.” However, there is a tendency to sideline or overlook the scale of capacity building needed as well as the larger bureaucratic challenges involved in fostering “country ownership” and institutionalising equitable and effective interventions within government ministries. Without sound project management that creates feedback loops and adaptive mechanisms between different actors (paying attention to embedded infrastructure, capacity and community participation issues), public health interventions like canine rabies vaccination will have difficulties in navigating local access barriers.

Addressing this requires time, leadership, resources, vision and institutional learning to effectively address the legacy of structural adjustment on the health and veterinary systems in developing countries and strengthen the relationship between the central government, district officials, extension workers and communities. Critical gaps between project planners, implementers and communities have also been noted, for example, in other recent studies on Neglected Tropical Disease control in Tanzania [40], [63]–[64]. Greater realisation that these issues need to be more proactively addressed is shown in contemporary emphasis on implementation research [47], systems-based approaches to infectious disease control (i.e. EcoHealth and One Health) [43]–[44] and the involvement of social scientists in NTD control [48], [65]. Understanding the context of success and failure, therefore, should be more encouraged by the NTD community if we are to learn from past experiences, propose future strategies and ultimately create more resilient and sustainable programmes, and more healthy communities.

An interesting example of how things can change on the ground and the need for flexibility and foresight in implementing a successful rabies elimination programme involves recent changes in dog populations in Kilombero and Ulanga since the end of field research. With the threat of environmental degradation in the fragile Kilombero Valley ecosystem, the government (with police support) forcibly evicted over 380,000 cattle in late 2012, likely the majority of pastoralists. As these cattle keepers now migrate to new districts, vaccination coverage in Kilombero and Ulanga will likely increase dramatically, but planning for future campaigns in the wider WHO elimination area will require consideration about where these livestock keepers, and their many dogs, have gone.
